# Ferroptosis landscape in prostate cancer from molecular and metabolic perspective

**DOI:** 10.1038/s41420-023-01430-0

**Published:** 2023-04-15

**Authors:** Jiaming Liang, Yihao Liao, Pu Wang, Kun Yang, Youzhi Wang, Keke Wang, Boqiang Zhong, Diansheng Zhou, Qian Cao, Junbo Li, Yang Zhao, Ning Jiang

**Affiliations:** 1 Tianjin institute of Urology, The Second Hospital of Tianjin Medical University, 300211 Tianjin, China; 2 School of Future Technology, Xi’an Jiaotong University, 710049 Xi’an, Shaanxi China; 3 Department of Urology, Tangdu Hospital, The Air Force Military Medical University, Xi’an, Shaanxi China; 4 Department of Radiology, Tianjin Medical University Second Hospital, Tianjin, China

**Keywords:** Prostate cancer, Cell death, Genetic interaction

## Abstract

Prostate cancer is a major disease that threatens men’s health. Its rapid progression, easy metastasis, and late castration resistance have brought obstacles to treatment. It is necessary to find new effective anticancer methods. Ferroptosis is a novel iron-dependent programmed cell death that plays a role in various cancers. Understanding how ferroptosis is regulated in prostate cancer will help us to use it as a new way to kill cancer cells. In this review, we summarize the regulation and role of ferroptosis in prostate cancer and the relationship with AR from the perspective of metabolism and molecular pathways. We also discuss the feasibility of ferroptosis in prostate cancer treatment and describe current limitations and prospects, providing a reference for future research and clinical application of ferroptosis.

## Facts

- Prostate cancer is second only to lung cancer in male incidence.
- There is no effective treatment for distant metastasis and castration resistance of advanced prostate cancer.
- The role of ferroptosis in prostate cancer has been verified, but there is a lack of summary at the metabolic level and the relationship between ferroptosis and AR.

## Open questions

- Could the interaction of ferroptosis and AR be the key to block the progression of prostate cancer with ferroptosis combination therapy?
- Can ferroptosis-related genes be used as specific biomarkers to judge the prognosis and immunotherapy sensitivity of prostate cancer?
- Is it possible to establish an individualized plan for phased ferroptosis combined therapy according to the patient’s metabolic level and tumor stage?

## Introduction

Prostate cancer (PCa) is the main disease that threatens the health of male urinary system [1]. PCa usually develops very slowly, and timely diagnosis can make its 5-year survival rate reach nearly 100% [2]. However, due to the inconspicuous symptoms in the early stage, the disease is usually only detected in the late stage or even in the advanced stage of metastases. In most cases, delays in detection and metastases of the disease make PCa treatment difficult, and only about 30% of cases have a survival prognosis of five years. Androgens can regulate tumor proliferation and are currently the main therapeutic targets in advanced PCa. Androgen deprivation therapy and anti-androgen therapy played a good role early in treatment. But with the development of resistance at a later stage, the impact of hormone therapy gradually weakens, causing further deterioration of the disease. Therefore, the search for new treatments as well as ways to diagnose the disease is inevitable.

The concept of iron death was first proposed in 2012 [3]. Subsequently, accumulating evidence has linked ferroptosis to several human diseases including PCa [4]. The discovery of ferroptosis sheds new light on cancer development. Tumor cells require more iron than normal cells due to their high metabolism, which also leads to an increase in the possibility of ferroptosis [5]. Ferroptosis inhibits tumor growth has been confirmed in many experiments, and the use of ferroptosis inducers and the regulation of related genes may become new weapons against cancer. Before this, it is necessary to find out the mechanism of ferroptosis in PCa.

## Metabolic and molecular regulators of ferroptosis

**Fig. 1:**
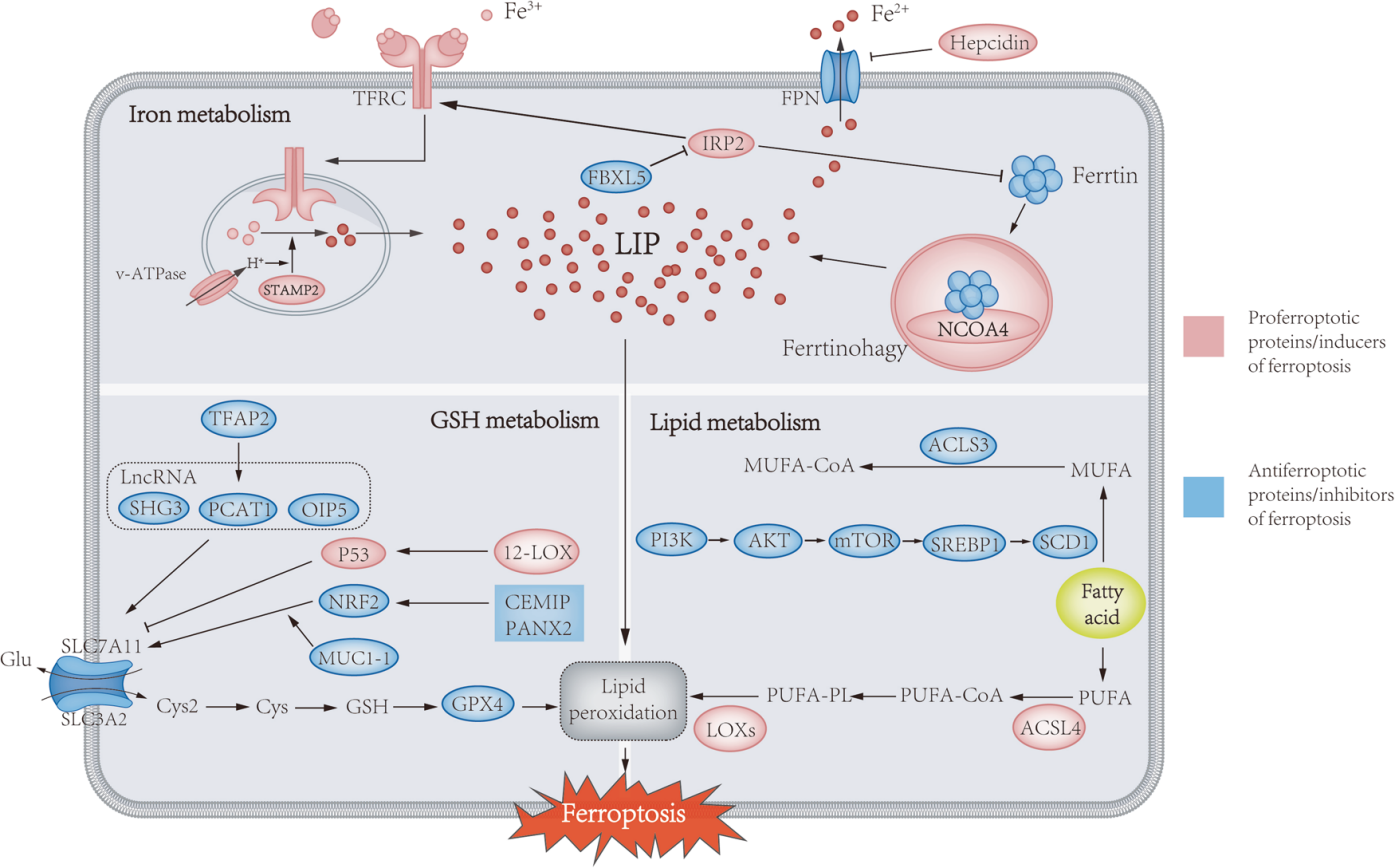
Metabolic pathways implicated in ferroptosis in prostate cancer. Ferroptosis is mainly caused by iron-related lipid peroxidation, and the absorption, storage, utilization and export of iron in iron metabolism will affect ferroptosis. STAMP2-mediated ferric iron reduction and NCOA4-mediated ferritin autophagy can increase labile iron pools to sensitize cells to ferroptosis via the Fenton response. The classical inhibitory pathway of ferritin metabolism is mainly uptake of Cys by the cystine-glutamate antiporter (system xc^−^), which is used for the synthesis of GSH. GPX4 utilizes GSH as a cofactor to reduce lipid hydroperoxides to lipid alcohols. In addition, the activation of ACSL4, LPLAT5, and LOXs in the lipid metabolism pathway can promote the peroxidation of PUFA and induce ferroptosis. Conversely, the PI3K/AKT/mTOR pathway is able to promote SREBP1/SCD1-mediated MUFA formation and convert MUFAs to their acyl-CoA esters under the action of ACSL3 for incorporation into membrane phospholipids, thereby protecting cancer cells from ferroptosis.

Unlike apoptosis, autophagy and necrosis, ferroptosis regulates cell death through iron-associated lipid peroxidation. Interestingly, the process of ferroptosis is unique among other forms of cell death in that it can occur in a wave-like pattern from one cell to another [6]. Cells undergoing ferroptosis have mitochondrial abnormalities in morphology, such as swelling, density changes, and outer membrane disruption, which can be observed by electron microscopy. The metabolism of iron, glutathione, and lipids contributes to ferroptosis and jointly control the initiation of ferroptosis. In the following, we will discuss the relationship between ferroptosis and PCa from a metabolic perspective (Fig. 1).

## Iron metabolism in PCa

Transferrin-mediated and lactoferrin-mediated iron uptake pathways are the main pathways for cells to take up iron, after binding to iron, the two proteins enter cells through corresponding receptor/non-receptor pathways [7]. In PCa, to provide cancer cells with more iron to metabolize and grow, TFR responsible for endocytic transferrin-bound iron is upregulated to increase iron uptake [8, 9]. As a downstream target gene of MYC oncogene, TFR can reflect the gene status of MYC, the combination of PET imaging and TFR-specific radiotracer has been successfully used to detect abnormal oncogene signals of precursors in PCa [10]. With MRI, TFR amplified with specific promoters can also be used to locate and visualize early PCa [11]. In PCa, the endocytosed transferrin entering cells through TFR is distributed throughout the cytoplasm and in cellular extensions, compared with non-malignant cells (which are mainly clustered in the perinuclear region) [12]. This altered endosome distribution may be a manifestation of malignant cells adapting to high iron requirements. The release of iron is dependent on the low pH of the endosome, which is mainly mediated by the V-ATPase. When V-ATPase activity decreases, iron release also reduces. The acidified endosome enables STAMP2 (six transmembrane epithelial antigen of the prostate 4) to reduce ferric irons to ferrous irons. Through its ferroreductase activity, STAMP2 increases the level of reactive oxygen species (ROS) in PCa cells while depleting NADPH levels, which accelerates the accumulation of ROS [13]. Stamp2 also regulates PCa cell proliferation by affecting mitochondria and autophagy in cancer cells [14]. As compared with other STEAP members, STEAP4 (STAMP2) plays a more important role in the regulation of iron metabolism in PCa. In addition, Ferritin can undergo selective autophagy mediated by nuclear receptor coactivator 4 (NCOA4) to promote ferroptosis by increasing labile iron in the cytoplasm [15].

Cellular iron export depends on ferroportin (FPN), as the only exporter of cellular iron identified so far, FPN is downregulated in PCa [16]. Through its effect on intracellular iron, FPN expression suppresses PCa cell proliferation independent of altered androgen sensitivity [17]. FPN can be inhibited by hepcidin. Hepcidin is significantly upregulated in PCa, acting in an autocrine manner to reduce iron excretion [18]. Like liver hepcidin, prostate hepcidin is also regulated by IL6 and BMPs (bone morphogenetic proteins). However, compared with the prominent role of BMP6 in liver hepcidin, BPM4/7 have a more pronounced role in regulating iron in the prostate. In addition, the wingless/integrated (Wnt) pathway also affects the expression of prostatic hepcidin, which differs in canonical pathways [18]. Hepcidin knockdown in PCA cells enhances cancer cell proliferation, migration and anti-apoptotic abilities through iron metabolism [19]. Hepcidin levels have also been found to correlate with cancer-associated fibroblast infiltration, its role in tumor immunology is worth exploring [20].

Iron regulatory proteins (IRPs) are important regulators of cellular iron metabolism, whose activity is affected by iron levels [21]. IRPs are primarily involved in iron regulation at the post-transcriptional level, IRP1 function is regulated by mitochondrial iron-sulfur (Fe-S) clusters, attachment on Fe-S prevents IRP1 from binding to other mRNAs for regulatory functions. As iron levels in the cells decrease, IRP1 dissociates from Fe-S clusters and binds to ribosomal entry sites. The activity and stability of IRP2 depend on its binding partner F-box and leucine-rich repeats protein 5 (FBXL5), which can degrade IRP2 by ubiquitination [22, 23]. In PCa, IRP2 is upregulated to accommodate increased iron requirements, overexpression of IRP2 results in upregulation of TFR and downregulation of ferritin. When IRP2 is knocked down, PCa cell growth and proliferation are inhibited in an iron-dependent manner. Compared with IRP2, IRP1 has less obvious effects on iron metabolism and PCa cell growth [24]. Although the reason for IRP2 overexpression in PCa is unclear, based on the importance of IRP2 in iron homeostasis, it could be a new effective target for cancer treatment.

## Glutathione metabolism in PCa

Glutathione (GSH) is a major antioxidant produced by the liver and consists of steine, glutamate, and glycine. As the preferred substrate of gpx4, depletion of GSH will reduces GPX4 activity and accelerates ferroptosis [25]. Cancer cells are subject to higher ROS oxidative stress than non-malignant cells due to altered growth and genetic mechanisms, and their growth and proliferation are inseparable from the antioxidant effect of GSH [26]. Using cysteinase deplete cysteine (Rate-limiting precursor for the synthesis of GSH) in mice, PCa xenograft growth is significantly inhibited [27]. Although cysteine can be produced by the endogenous pathway, most of it is obtained by the system xc^-^, which can absorb cystine into cells and excrete glutamate 1:1 [28]. The cystine transported into the cell is reduced to cysteine by consumption of NADPH [29]. The system xc^-^ consists of 2 subunits, solute carrier family 3 member 2 (SLC3A2; also known as 4F2hc) and solute carrier family 7 member 11 (SLC7A11; also known as xCT). SLC7A11 is a transmembrane protein, mainly involved in the antiport of cystine/glutamate on the plasma membrane, while SLC3A2 mainly acts as a chaperone to stabilize SLC7A11 and help protein complex localize to the membrane [30, 31].

The expression of SLC7A11 in PCa is increased compared with normal, which may be a strategy to combat ferroptosis [32]. The effect of erastin (a ferroptosis inducer) was found to be more pronounced in SLC7A11 knockout PCa cells compared to controls [33]. There is a positive correlation between SLC7A11 and NRF2 [34]. CEMIP (cell migration-inducing protein) can elevate the level of SLC7A11 in PCa cells by promoting NRF2 phosphorylation and nuclear localization, thereby promoting cellular ferroptosis resistance [35]. MUC1-1cy can interact with NRF2 and PRM1 on the SLC7A11 promoter to regulate SLC7A11, and silencing MUC1-1 reduces the occupancy and chromatin accessibility of NRF2 and PRM1. Through this pathway, MUC1-1 regulates redox balance and oxidative stress in PCa cells [36]. Liao et al. found that silencing PANX2 (a member of the pannexin family) can promote ferroptosis by downregulating NRF2 to inhibit the progression of PCa, and the inhibitory effect of PANX2 silencing on PCa was reversed after NRF2 activation with oltipraz [37]. NRF1 and NRF2 are structurally and functionally related, and the loss of either can be complemented by overexpression of the other in terms of ferroptosis sensitivity. Interestingly, NRF1 can reduce ferroptosis sensitivity by maintaining the expression of GPX4, and this effect is independent of NRF2 [38]. Conversely, p53 represses SLC7A11 transcription [39]. Flubendazole, an antimalarial drug, was found to promote apoptosis in PCa cells by inducing p53 expression thereby inhibiting SLC7A11 transcription and activating ferroptosis [40]. In addition, some lncRNAs (such as PCAT1 [41], SNHG3 [42], OIP5-AS1 [43]) have also been shown to participate in the regulation of PCa cell metabolism by regulating SLC7A11, in which PCAT1 is transcriptionally positively regulated by transcription factor AP-2 gamma (TFAP2C) to increase ferroptosis resistance [41]. As the most upstream regulator of ferroptosis, SLC7A11 plays an important role in ferroptosis. This makes SLC7A11 the key to ferroptosis-related treatment in PCa.

The antioxidant enzyme GPX4, a selenocysteine-containing enzyme that blocks iron-mediated peroxidation by eliminating lipid peroxides (LPO) and thus protects cell membranes from damage. The excessive expression of GPX4 can inhibit RSL3-induced iron death, and absence of GPX4 will reduce the resistence to ferroptosis [44]. ChaC glutathione specific gamma‑glutamylcyclotransferase 1 (CHAC1), a proapoptotic endoplasmic reticulum stress protein, was found to correlate the viability of PCa cells and GSH levels. The increase of LPO level and the decrease of GPX4 level can be observed in CHAC1 overexpressed cells. In addition, the CHAC1 protein will be expressed more strongly following treatment with ferroptosis activator, indicating a key role for CHAC1 in PCa ferroptosis [45]. As a selenoprotein, the synthesis of GPX4 is dependent on the concentration of selenium [46]. Selenium is required for GPX4 synthesis, and selenium addition to prostate normal/cancer cells increases GPX4 expression and prevents cellular oxidative stress. Interestingly, PCa cell did not express a higher sensitivity to selenium compared to normal prostate epithelial cells, instead, normal prostate epithelial cells had higher utilization of selenium. This reduced selenium utilization by cancer cells may explain the reduced expression of selenoproteins during carcinogenesis [47]. Despite numerous studies exploring selenium’s role in PCa, there is no consensus on this, and the results obtained by these studies have shown inconsistency (Table 1) [48–54]. Individual differences in experimental subjects may be the main reason for this phenomenon, Karunasinghe and Martinez et al. showed levels of specific genes affects the relationship between selenium and PCa [55, 56]. Moreover, the initial amount of selenium content in the human body is also an important factor. The effect of selenium supplementation on preventing PCa is more obvious in selenium-deficient people than in selenium-enriched people [57]. This also explains why the results of the study have changed over time, possibly due to the improvement of people’s quality of life, the transition of selenium levels in the body from deficiency to enrichment, which downplays the effect of selenium on the prevention of PCa. To be sure, selenium metabolism plays an important role both in the development of prostate cancer and in ferroptosis [58].

**Table 1:** Effects of selenium on prostate cancer.

Study	Year	Source	Numbed of patients	Age (years)	Follow-up (years)	Findings	References
Randomized controlled trial	2011	USA	423	>40	3	Selenium benefited selenium-deficient men but did not prevent PCa in selenium-replete men	[57]
Randomized controlled trial	2009	USA	35,533	>50	5.5	Selenium did not prevent PCa in the generally healthy, heterogeneous population of men	[48]
Randomized controlled trial	2013	USA	699	<80	5	Selenium had no significant effect on PCa incidence and PSA velocity	[49]
Randomized controlled trial	1998	USA	974	NA	6.5	Selenium intake significantly reduced PCa risk	[50]
Case–control	1998	USA	51,529	40–75	8	Higher selenium levels were associated with a reduced risk of advanced PCa	[51]
Randomized controlled trial	2019	New Zealand	572	20–80	0.5	Significant negative correlation between selenium changes and PSA changes in men below the median age, never smoke, carrying the GPX1 rs1050450 T allele, dietary intakes above the recommended daily intake (RDI) for zinc, and below the RDI for vitamin B12	[55]
Randomized controlled trial	1996	USA	1312	18–80	6.4	Selenium supplementation was associated with significant reduction in PCa incidence and mortality	[52]
Randomized controlled trial	2014	USA	4856	>50	5.5	Selenium supplementation did not benefit men with low selenium status but increased the risk of high-grade PCa among men with high selenium status	[53]
Randomized controlled trial	2014	USA	5001	>50	5.5	The link between PCa risk and selenium supplementation could be modified by NKX3.1 genotype	[56]
Mendelian randomization analysis	2018	PRACTICAL Consortium	72,729	NA	NA	Selenium supplementation may adversely affect the risk of advanced prostate cancer	[54]

The extensive physiological functions of GSH make it a factor that cannot be ignored in the occurrence and development of PCa. GSH depletion is inevitable during aging and commonly occurs in tissues and blood of aging organisms, which also increases damage associated with oxidative stress. Due to the inhibitory role of GSH in carcinogenesis, GSH depletion may in part contribute to increased cancer risk [59, 60]. A meta-analysis study based on PCa metabolic levels found that PCa patients had lower GSH levels than healthy controls [61]. In mice fed with a cystine-rich diet, increased GSH levels in the prostate epithelium were observed, which inhibited PCa progression by enhancing the cysteine-GSH antioxidant system [62]. Interestingly, the researchers found that more aggressive PCC had higher GSH levels compared to less aggressive [63]. Therefore, a stratified analysis may be required to analyze the relationship between GSH and PCa. In noncancer people, GSH has a preventive effect on them, but in cancer-people, GSH provides cancer cells with protection against oxidative stress, further enhancing the aggressiveness of cancer cells. This may provide a staged prevention and treatment program for PCa in the future.

## Lipid metabolism in PCa

Phospholipids containing PUFAs in cell membranes are highly sensitive to lipid peroxidation, which is critical for ferroptosis [64]. Therefore, the content of PUFA in the cell membrane is an important factor affecting ferroptosis. PLA2G4A, an enzymatic member of the A2 family IV family of cytosolic phospholipases, metabolizes arachidonic acid (AA) to eicosanoid, and PLA2G4A is regulated by transcription factor 6α (ATF6α), which can accelerate arachidonic acid metabolism by upregulating PLA2G4A to avoid ferroptosis in cancer cells [65]. The Acyl-CoA synthetase long-chain family member 4 (ACSL4) activates PUFAs to form long-chain acyl-CoAs and attaches to the cell membrane, which powers ferroptosis because it increases the ratio of polyunsaturated fatty acids in the membrane, making cells more susceptible to ferroptosis [66, 67]. The ACSL4 expression is up-regulated in several types of cancers (including ER-negative breast, colorectal and prostate cancers) and down-regulated in others (ER-positive breast, lung and cervical cancers). ACSL4 positively correlates with PCa cell proliferation, migration and invasion, and this correlation exists both in vivo and in vitro. A significant increase in ACSL4 was also observed when entering the castration-resistant phase, suggesting that ACSL4 expression may contribute to castration-resistant prostate cancer (CRPC) [68]. Furthermore, the use of inhibitors targeting ACSL4 reduced prostate tumor growth, resistance to related treatments, and steroid production, confirming this speculation [69]. By contrast, ACSL3, mainly located in the brain, prostate and muscle, is responsible for the activation and conversion of monounsaturated fatty acids (MUFA) into fatty acyl-CoA, competitively inhibiting PUFA-associated ferroptosis by altering the proportion of cell membrane components [70–72]. In addition, ACSL3 can promote the development of CRPC by increasing steroid production [73]. In nude mice, ACSL3 can promote tumor growth, and high expression of ACSL3 in PCa patients also predicts poorer prognosis [74]. As a rate-limiting enzyme, stearoyl-coenzyme A desaturase 1 (SCD1) can inhibit ferroptosis by converting saturated fats acids to monounsaturated fats acids (MUFA). Yi et al. found that silencing of sterol regulatory element-binding proteins 1 (SREBP1) reduced SCD1 expression to promote lipid peroxidation and induce ferroptosis in PCa cells [75]. In addition, the researchers also found that the PI3K/AKT/mTOR pathway can inhibit ferroptosis through the SREBP1-SCD1 axis, but the relationship between the PI3K/AKT/mTOR pathway and PCa in terms of ferroptosis remains unclear. 2,4-Dienoyl-CoA reductase 1 (DECR1) can limit the rate of PUFA oxidation, and its expression is significantly increased in prostate tumors and castration-resistant mice, but the opposite is observed in breast cancer [76, 77]. DECR1 knockdown selectively inhibits PUFA β-oxidation leading to cellular accumulation of PUFA, leading to excess accumulation of PUFA in cells, providing raw material for oxidative stress and ferroptosis [78].

Lipoxygenase, a non-heme iron- or manganese-containing oxygenase with the ability to oxidize polyunsaturated fatty acids and their derivatives in cell membranes, plays a key role in ferroptosis [79]. In PCa, 5-Lipoxygenase (5-LOX), 12-Lipoxygenase (12-LOX), and 15-Lipoxygenase (15-LOX) have always been the research hotspots. Numerous studies have shown that 5-LOX can be a target of ferroptosis, and inhibition of 5-LOX can reduce the risk of cellular ferroptosis by neutralizing lipid peroxidation [80]. 5-LOX can combine with AA to generate 5-HETE (5-hydroxyeicosatetraenoic acid), which is a potent survival factor for PCa cells. When the formation of 5-HETE is blocked, PCa cells undergo apoptosis in a very short time, which can be rescued by exogenous 5-HETE [81]. 12-LOX is upregulated in PCa, and it stimulates the growth and generation of tumor blood vessels to promote PCa progression [82]. Interestingly, as a member of the lipoxygenase family, in addition to its function of oxidizing PUFAs, 12-LOX induces P53-mediated ferroptosis, and this pathway is ACSL4-independent, which is essential for p53-dependent tumor suppression [83]. 15-LOX2, as the main lipoxygenase in normal prostate, has the function of negatively regulating the cell cycle of prostate epithelial cells and can inhibit the occurrence of prostate cancer. When normal cells undergo malignant transformation, its expression level will decrease [84]. 15-LOX can bind to and oxidize PUFAs in the membrane under the guidance of PEBP1 (Protein Kinase Cascade Inhibitor), thereby promoting ferroptosis [85].

The imbalance of the antioxidant system will cause the oxidation of PUFA and the accumulation of LPO, which will trigger a series of pathological or physiological changes. To detect this process, lipid peroxidation is usually labeled with specific electrophilic reactive aldehydes (end products of PUFA oxidation) such as malondialdehyde, 4-hydroxyhexenal, and 4-hydroxynonenal [86]. Comparing the oxidative and antioxidant levels in the prostate of young (2-month-old) and aged (9-month-old) rats, it was found that the aged group had lower antioxidant levels and higher oxidative damage, which may be caused by aging [87]. Interestingly, rats with higher levels of oxidative stress developed prostate hyperplasia and cancer more frequently, which may be part of the reason why PCa occurs more frequently in older age [88]. In addition, a survey study found that serum malondialdehyde levels in PCa patients were significantly higher than in healthy controls [89]. Together, these studies suggest that lipid peroxidation plays a non-negligible role in the initiation and progression of PCa.

## Metabolic alterations during PCa progression

To meet the needs of growth and development, tumor cells undergo a series of metabolic shifts, but unlike other tumors, PCa cells tend to enhance oxidative phosphorylation rather than glycolysis at an early stage [90]. This metabolic reprogramming is associated with the regulation of AR and the loss of zinc transporters during carcinogenesis [91, 92]. When the disease progresses to CRPC, in addition to changes in AR signaling, factors such as loss of p53 and PTEN, activation of GR and STAT, upregulation of MYC and BUB1, and mutations of PI3K, BRAC1, and BRAC2 further contribute to the metabolic heterogeneity in CRPC [93, 94]. At this stage, the reactivation of AR signaling can tilt the metabolic pattern towards glycolysis by increasing GLUT1 (glucose transporter) [95]. If bone metastases occur, bone marrow adipocytes are able to activate glycolytic enzymes in CRPC cells to synergistically drive the Warburg effect with AR [96]. Furthermore, the formation of AR splice variants in CRPC enables the activation of key enzymes such as FASN to re-engage in de novo lipogenesis [96]. The enhancement of glucose metabolism in CRPC brings the anti-oxidative raw material glutamine to prevent ferroptosis, but at the same time, the accumulation of fat content increases the risk of lipid peroxidation.

Neuroendocrine prostate cancer (NEPC) is a deadly subtype of prostate cancer that is often seen in CRPC therapy and presents enormous difficulties for treatment [97]. Unlike PCA, which is usually adenocarcinoma, NEPC tumors lose their dependence on AR signaling, and the regulation of many metabolic processes by AR is correspondingly reduced, the activation of related signaling pathways and chromatin modifications lead to metabolic reprogramming [98]. Wang et al. find that activation of EZH2 and KDM8 drives NEPC metabolic programming to aerobic glycolysis [99]. This conclusion was verified by Choi et al., who found that NEPC cell proliferation decreased after targeted inhibition of glucose metabolism in a xenograft model [100]. In terms of lipid metabolism, in addition to classical lipogenesis and β-oxidation, the lipid coordination pathway mediated by Apolipoprotein A-I (apoA-I) is highly activated [101]. Uncovering and exploiting changes in these metabolic profiles may inform the introduction and timing of ferroptosis therapy.

## The role of AR in ferroptosis in PCa

**Fig. 2:**
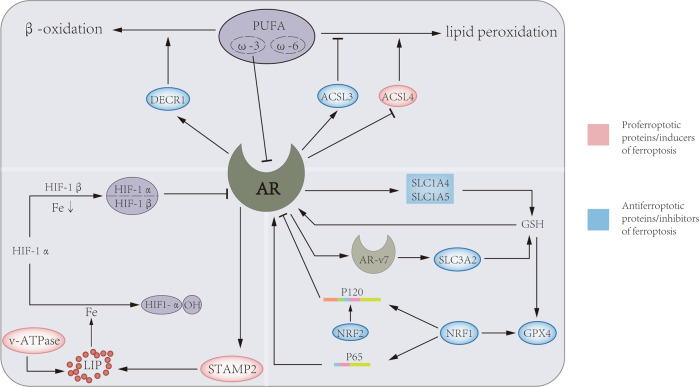
The relationship between AR and ferroptosis. AR can regulate three metabolic pathways in ferroptosis. On the one hand, AR regulate DECR1 to promote the β-oxidation of PUFA, and regulate the lipid composition in the cell membrane through ACSL3 and ACSL4. On the other hand, AR and its mutant AR-V7 increase oxidative resistance by upregulating the level of GPX4 through GSH metabolism mediated by SLC1A4, SLC1A5 and SLC3A2, respectively. Furthermore, AR can promote STAMP2-mediated redox reactions and increase ferrous iron levels in the cytoplasm. At the same time, iron, GSH, and PUFA can also affect the level of AR through different ways.

The androgen receptor (AR), as a steroid hormone receptor, can activate the transcription of androgen target cells in a ligand-dependent manner, and is an important driving factor for the progression of prostate cancer. In recent years, the regulatory relationship of AR in ferroptosis has been gradually discovered (Fig. 2). In iron metabolism, AR increases the expression of STAMP2, which reduces Fe^3+^ to Fe^2+^ through its ferroreductase activity while depleting available NADPH. Increased Fe^2+^ and decreased NADPH lead to increased intracellular ROS levels [13]. In lipid metabolism, androgens were able to increase the uptake of fatty acids, cholesterol, and lipoproteins in AR-positive PCa cell lines, which can be blocked by AR antagonists. The expression levels of ACSL3 and ACSL4 are inversely regulated by androgen-AR signaling, and activation of AR signaling significantly upregulates ACSL3 levels and decreases ACSL4 levels, thereby maintaining AR-dependent and AR-independent fatty acid metabolism in PCa cells. It is worth noting that the regulation of ACSL4 transcription by AR is very weak, but the higher catalytic efficiency of ACSL4 compared with ACSL3 makes up for this defect, so the biosynthesis of fatty acyl-CoA can be well maintained [102]. An important enzyme for lipid oxidation, DECR1, is a target gene downstream of AR, through which AR can regulate intracellular PUFA levels, thereby affecting ferroptosis [78]. In GSH metabolism, AR signaling can promote glutamine metabolism by regulating glutamine transport mediated by SLC1A4 and SLC1A5 [103]. In addition, AR-V7 (a splice variant of AR), which can affect system xc^-^ by regulating its downstream target gene SLC3A2, and further regulate amino acid transport [104].

The relationship between AR and ferroptosis is reciprocal, AR is also affected by some of these factors while regulating ferroptosis. Selenite can downregulate AR expression by depleting GSH, and it can be reversed by adding GSH, which illustrated the positive regulatory relationship of GSH to AR [105]. PUFAs can be mainly divided into two series of ω-3 and ω-6 according to their structural characteristics and functions. ω-6 PUFAs can promote the growth of PCa cells, and a high ω-6 PUFAs-diet will increase the risk of PCa. Conversely, ω-3 PUFAs can inhibit the growth of prostate tumors by reducing the expression of AR through the reduction of testosterone and estradiol levels [106]. In ferroptosis, NRF1 and NRF2 are able to coordinate with each other to maintain cellular homeostasis, and this relationship persists in regulating AR activity [107]. The human Nrf1 gene produces full-length 120 kDa NRF1 (p120-NRF1) and several truncated (36, 55, 65, and 95 kDa) NRF1 isoforms. Among them, p65-NRF1 can interact with AR to form a complex and induce AR transactivation, while p120-NRF1 can inhibit this effect by competing with p65-NRF1 for AR binding. The ratio of p65-NRF1 to p120-NRF1 in PCa cells determines their impact on AR activity, and this ratio is regulated by NRF2, which promotes p120-Nrf1 accumulation in the nucleus and reduces AR transactivation [108]. The balance of NRF1 and NRF2 may be the key to the progression of PCa. After treatment of androgen-dependent PCa cells (LNCaP) and castration-resistant PCa cells (C4-2) with dihydrotestosterone (DHT), the transactivation of AR by DHT was found to be more pronounced in LNCAP cells. Mechanistically, DHT increased p65-NRF1 nuclear translocation and suppressed NRF2 levels in LNCAP cells, but increased NRF2 levels in C4-2 cells [90]. This suggests that castration resistance may be caused by a tilt in the balance of NRF1 and NRF2. V-ATPase can regulate the expression of AR through the mediation of HIF-1α, and this relationship still exists even if AR is mutated. HIF-1α requires iron as a cofactor for hydroxylation to HIF-1α-OH. When V-ATPase is inhibited, the release of iron to the cytoplasm is reduced, resulting in an iron-deficient state of the cell. HIF-1α will combine with HIF-1β to become HIF-1α/β, which can combine with the AR gene promoter to reduce AR expression [109, 110].

## Other factors regulating ferroptosis

Similar to glutamine, the metabolism of branched-chain amino acids (BCAAs) can also affect ferroptosis. High concentration of BCAA can promote ROS generation and damage mitochondrial function through Akt-mTOR pathway [111]. The BCAA ingested by the human body is catabolized by branched-chain amino acid transaminase (BCATs), which have two isoforms, BCAT1 and BCAT2 [112]. Interestingly, BCAT1 and BCAT2 were shown to protect cells from ferroptosis by up-regulating GSH and activating the system xc^-^, respectively, which were opposite under high-concentration BCAA conditions [113, 114]. In addition, some amino acids such as tryptophan (Trp) and arginine (Arg) have also been shown to be involved in the regulation of ferroptosis. The metabolite of Trp, indole-3-pyruvate (I3P), can activate the anti-oxidative stress system by promoting the transcription of SCL7A11. Not only that, I3P itself also has the ability to scavenge free radicals. Both ways work together to prevent ferroptosis [115]. The regulation of Arg on ferroptosis may be realized through GPX. The study found that the addition of Arg can increase the level of GPX in cells while reducing the level of ROS and malondialdehyde [116].

Ferroptosis inhibitory protein 1 (FSP1), as a glutathione-independent anti-ferroptosis factor, can catalyze the production of coenzyme Q_10_, which has antioxidant properties [117]. At present, there is a lack of research on FSP1 in PCa, and the verification of coenzyme Q10 in oxidative stress in PCa is still stagnant in the study done by Quiles et al. in 2003, which showed that compared with malignant cells, the antioxidant effect of coenzyme Q_10_ is more obvious in non-malignant cells [118]. For the entire PCa ferroptosis regulatory network, the FSP1-CoQ10 axis cannot be ignored, and it is worth exploring its mechanism.

## The role of ferroptosis in bone metastasis of PCa

Compared with primary PCa, the level of oxidative damage in PCa bone metastases cells is elevated, suggesting that the latter is responsible for higher oxidative stress [119]. Interestingly, the content of antioxidants and thiol compounds in PCa bone metastases also increased accordingly [120]. This enhanced antioxidant capacity seems to be an adaptation of cells to respond to changes in oxidative levels and also reflects the importance of ferroptosis in PCa bone metastases. The high metabolic activity of cancer cells makes it necessary to synthesize more GSH to prevent oxidative stress, and the level of glutamate, which is the raw material for synthesis, also increases [121, 122]. As a communication molecule between normal skeletal cells, excess glutamate may alter bone metabolic homeostasis through receptor-dependent mechanisms. The activation of glutamate receptors is important in osteoclast differentiation, thus alterations in glutamate levels may cause disruption of the bone resorption process [123]. A series of bone changes provide favorable conditions for bone metastasis.

The bone microenvironment acts as an important mediator in the process of PCa cell metastasis to bone, which can affect the transition between the dormant and proliferative states of cancer cells [124]. High free fatty acid levels in patients induce bone mesenchymal stem cells to differentiate into adipocytes rather than osteoblasts, thereby altering the bone microenvironment and promoting bone metastasis of PCa [125]. PUFA can induce the differentiation of bone marrow adipocytes, among which AA is the most obvious and AA is also the main raw material of LPO. Bone marrow adipocytes can interact with cancer cells, prompting cancer cells to directly uptake and metabolize AA, destroy adipocytes and subsequently form bone metastases [126]. In SCID mice, when 12-LOX-transfected PCa cells were injected via the tail vein into mice implanted with human bone fragments, increased metastasis of cancer cells transfected with 12-LOX to human bone compared with control vector-transfected cells, suggesting that overexpression of 12-LOX enhanced the metastatic potential of PCa cells [127].

The appropriate iron level can maintain the stability of the bone environment, and excessive or insufficient iron content will disrupt the balance of bone turnover by affecting the differentiation of osteoblasts and osteoclasts [128]. STAMP2, as the only upregulated protein of the STEAP family in osteoclast differentiation, is involved in the iron uptake of osteoclast precursors and the generation of reactive oxygen species, which are required for the formation and function of mature osteoclasts [129]. In the femurs of mice implanted with PCa cells, changes in trace elements were observed, indicating osteolysis in the bone tissue. Adding iron supplements to the diets of these mice slowed the changes in these elements, suggesting that iron may inhibit PCa bone metastases by improving bone composition [130]. β2-M and its receptor hemochromatosis (HFE) protein play an important role in the proliferation and bone metastasis of PCa [131]. The complex of the two can bind to transferrin and reduce transferrin and transferrin receptors affinity to prevent the occurrence of iron overload [132]. Inhibition of β2-M or HFE increases iron and ROS levels in PCa cells, promotes ferroptosis, inhibits tumor growth in a bone xenograft model, and upregulates sensitivity to chemoradiotherapy [133].

## Ferroptosis as a promising treatment in PCa

### Ferroptosis inducers

Given its role in the prostate, ferroptosis is a promising strategy for treating PCa. In recent years, people have discovered many different mechanisms of ferroptosis inducers, and confirmed the inhibitory effect of ferroptosis inducers in PCa (Table 2) [134–145].

**Table 2:** Application and mechanism of ferroptosis inducers.

Ferroptosis inducers	Mechanisms	Phenotype	References
Erastin	Inhibit System xc^−^	ROS accumulation, lipid peroxidation, GSH depletion, SLC7A11 upregulation	[134]
Sulfasalazine	Inhibit System xc−	Lipid peroxidation, GSH depletion, SLC7A11 upregulation	[135, 136]
Sorafenib	Inhibit System xc−	Increased overall ROS level and mitochondrial ROS level, lipid peroxidation, GSH depletion	[137, 138]
RSL3	Inhibit GPX4	ROS accumulation, lipid peroxidation	[44]
Diallyl trisulfide	Inhibit GPX4	ROS accumulation, increase the labile iron pool	[149]
6-Gingerol	Inhibit GPX4	ROS accumulation, GSH depletion	[150]
Altretamine	Inhibit GPX4	Lipid peroxidation	[139, 144]
Dihydroartemisinin	Inhibit GPX4, elevate Fe^2+^ level	Increased overall ROS level and mitochondrial ROS level, lipid peroxidation, GSH depletion, decreased GPX4 and ATP level	[145]
FIN56	Degrade GPX4	Lipid peroxidation	[141]
DPI2	GSH depletion	Lipid peroxidation	[44]
Cisplatin	GSH depletion	ROS accumulation	[142]
Buthionine sulfoximine	GSH depletion	Lipid peroxidation, GSH depletion	[44]
Artesunate	Elevate Fe^2+^ level, GSH depletion	ROS accumulation, lipid peroxidation	[143, 148]
Flubendazole	Induce p53 express	ROS accumulation, decreased SLC7A11 and GPX4 level	[40]

Enzalutamide and abiraterone, the second-generation AR antagonists, are commonly used drugs in the treatment of advanced PCa. Recent studies found that the combination of enzalutamide or abiraterone with erastin and RSL3 was more effective in suppressing PCa tumors than when they were used alone. Mechanistically, enzalutamide can reduce the expression of heat shock proteins (HSP) to reduce the resistance to ferroptosis, thereby synergizing with ferroptosis inducers to exert curative effect [4]. On the other hand, erastin may also down-regulate the expression of AR through the GSH-mediated pathway, thereby producing a synergistic effect with enzalutamide or abiraterone [105, 146].

Besides these common inducers of ferroptosis, some novel factors have also been shown to induce ferroptosis. The antimalarial drug flubendazole was recently found to inhibit SLC7A11 transcription by activating p53 expression to further down-regulate GPX4 levels and induce ferroptotic cell death in PCa cells [40]. Androgens play a driving role in PCa development. However, paradoxical inhibitory effects were observed in PCa cells when supraphysiological testosterone was administered. Subsequent studies have found that this inhibition is partly through supraphysiological testosterone-induced ferritinophagy, the degradation of ferritin disrupts cellular iron homeostasis and triggers ferroptosis [147]. The regulatory function of androgens on lipid metabolism may also be involved in promoting ferroptosis, which requires further research to prove.

In nature, specific components in some herbal remedies have also been shown to affect ferroptosis. Artesunate (artemisinin derivative) [148], diallyl trisulfide (garlic derivative) [149], 6-gingerol (turmeric extract) [150] can all up-regulate the ROS level of cancer cells and induce ferroptosis. Among them, artesunate is not only able to inhibit treatment-sensitive PCa cells, but also effective in docetaxel (DX)-resistant cancer cells. However, whether artesunate has a reversing effect on cancer cell resistance remains unclear. In advanced PCa, ART may be expected to be used as a supplementary treatment together with conventional treatment. Besides, scientists have artificially synthesized some ferroptosis inducers. 4-methyl-2-(4-methylpiperazinyl)pyrimido[4,5-b]benzothiazine (4-MMPB) and its two analogs, 4-propyl-2-(4-methylpiperazinyl)pyrimido[4,5-b]benzothiazine (4-PMPB) and 4-ethyl-2-(4-methylpiperazinyl)pyrimido[4,5-b]benzothiazine (4-EMPB) are synthetic compounds thought to have antineoplastic properties in PCa, which is achieved by promoting ferroptosis [151]. Qin et al. synthesized isothiocyanate-modified AR antagonists, which showed synergistic activity when combined with buthionine sulfoximine to treat CRPC cells, and resulted in the downregulation of AR/AR-V7 and ferroptosis [152].

Ferroptosis can be affected by externally adding ferroptosis-related metabolic substrates. The addition of iron and docosahexaenoic acid (DHA) can induce ferroptosis in PCa cells by affecting the balance of cellular iron metabolism and lipid metabolism, respectively [153, 154]. More importantly, the addition of iron potentiated the efficacy of antiandrogenic drugs, and in PCa xenografts, the combination of bicalutamide and iron exacerbated the oxidative damage of cells and inhibited the growth of tumors, which was not the case when the two were used alone. The addition of ferroptosis may become a new partner to amplify the efficacy of antiandrogens.

### Ferroptosis and drug resistance

In the process of drug treatment, PCa cells may undergo neuroendocrine transdifferentiation and thus acquire drug resistance. Along with cell differentiation, the activity of polyunsaturated lipid synthases is also upregulated, which provide conditions for lipid peroxidation. This alteration in lipid metabolism makes drug-resistant cancer cells more dependent on GPX4 and more prone to ferroptosis [155]. Taking advantage of this property, Chen et al. designed a biomimetic tumor-targeting magnetic lipid nanoparticle (t-ML) to deliver PUFA to drug-resistant cancer cells under the premise of knocking down DECR1 levels in drug-resistant cancer cells, effectively inducing ferroptosis. More importantly, in mice with tumor metastases, the use of t-ML significantly inhibited tumor growth without distant organ metastasis [156]. Wang et al. constructed an nanoplatform, PSMA-targeted arsenic nanosheets (PMANs), which can not only reduce the production of GSH by inhibiting the expression of SLC7A11 and GPX4, but also promote the consumption of GSH, and significantly increase the levels of intracellular ROS and LPO. In addition, the large surface area enables PMANs to transport doxorubicin (DOX) to PCa for synergistic therapy and sensitize cancer cells to DOX [157].

In addition to inducing ferroptosis, ferroptosis inducers also have an effect on drug resistance. RSL3 can induce ferroptosis by inactivating GPX4, and studies have found that RSL3 can also induce glycolytic dysfunction in PCa cells by reducing ATP and pyruvate content and protein levels of PKM2, PFKP, and HKII, which can be rescued by supplement of exterior sodium. More importantly, this effect of RSL3 on cellular glycolysis can enhance the sensitivity of PCa to cisplatin, and in vivo studies, combined treatment of low-dose RSL3 and cisplatin significantly reduces PCa expansion with no significant adverse effects [158]. In DX-resistant PCa cells, DHA can induce ferroptosis and reverse drug resistance by regulating PI3K/AKT/NRF2/GPX4 signaling pathway. More importantly, DHA can also inhibit Glutathione-S-transferase π through PI3K/AKT signaling pathway to reverse cell drug resistance [159].

In PCa cells, some components can act as a “bridge” between ferroptosis and drug resistance and function in both. CHAC1 promotes ferroptosis by upregulating LPO levels and downregulating GPX4 levels. The addition of docetaxel (DTX) along with plasmid overexpression of CHAC1 significantly reduced cancer cell viability, whereas docetaxel alone had a negligible effect [45]. Docetaxel-resistant PCa cells were also resistant to ferroptosis, which may be due to the high expression of PCAT1 in cells, which can interacts with c-Myc and competes with miR-25-3p to stimulate SLC7A11 to promote chemoresistance, and when PCAT1 is knocked down, cell resistance to both docetaxel and ferroptosis is attenuated [41]. Therapies that prompt immune cells to kill tumor cells by blocking the PD-1/PD-L1 pathway have shown good results in a variety of cancers, but have had little success in PCa. Recent studies have found that heterogeneous ribonucleoprotein L (HnRNPL) may be the key to breaking the embarrassing status of immunotherapy in PCa. Knocking out HnRNPL can down-regulate the expression of PD-1 while enhancing the induction effect of activated T cells on ferroptosis, increasing the sensitivity of cancer cells to T cells so that immunotherapy can effectively kill PCa cells [160].

### Ferroptosis-related genes as biomarkers

Wang et al. [161], Liu et al. [162], and Lu et al. [163] constructed different prostate cancer risk prediction models based on four (E2F1, CDC20, TYMS and NUP85), seven (AKR1C3, ALOXE3, ATP5MC3, CARS1, MT1G, PTGS2 and TFRC), and nine (AIFM2, AKR1C1, AKR1C2, CBS, FANCD2, FTH1, G6PD, NFS1 and SLC1A5) ferroptosis-related genes (FRG), respectively. The results showed that high FRG scores patients had poorer clinicopathological features, higher likelihood of biochemical recurrence and worse prognosis. Surprisingly, the FRG score was positively correlated with tumor mutation burden, which was associated with immunotherapy sensitivity, bringing a turn for the so-called “immune desert” of PCa. FRG score can be used as a new tool to distinguish PCa high/low risk groups and a potential prognostic indicator of immunotherapy outcome. However, the accuracy of its prediction still needs clinical trials to verify and more related mechanism studies are needed to provide a theoretical basis for it.

## Limitation

The important role of ferroptosis in PCa has been proved by more and more studies. However, there are still some issues that need to be clarified before ferroptosis therapy can be introduced into the clinic. First, the key mechanisms of ferroptosis in PCa need to be revealed. In recent years, the mechanism of ferroptosis has been broadly understood but lacks validation in PCa. For example, mitochondria, as energy powerhouses in eukaryotic cells, powering the initiation of ferroptosis, but there is a lack of related experiments in PCa, which casts a veil on the mechanism of ferroptosis. Second, there is a lack of reliable FRG as biomarkers. Although studies have found that FRG levels can reflect the prognosis of prostate cancer and the sensitivity of immunotherapy to a certain extent, the expression of these genes may be affected by the relevant metabolic levels of the individual, leading to misjudgment of the disease, so specific ferroptosis-specific biomarkers are crucial for clinical application of ferroptosis. Finally, is it possible to design an individualized protocol for the clinical application of ferroptosis? Metabolism related to ferroptosis will be affected by individual factors such as age, gender, diet, and genetic differences. At the same time, in different stages of PCa progression, tumor metabolism levels are also different, which will affect the efficacy of ferroptosis therapy. Therefore, the diagnosis and treatment plan for clinical application of ferroptosis must be individualized and phased, and the combination of related drugs also needs to be selected according to the patient’s condition.

## Conclusions

The correlation between iron and prostate cancer was first proposed nearly 40 years ago, and then the emergence of the concept of ferroptosis successfully revealed the mechanism behind this relationship, provided a new idea and perspective for cancer research, and also brought a glimmer of hope for clinical cancer treatment. In this article, we summarize the regulation of ferroptosis in PCa at the metabolic level, and analyze the application prospects and limitations of ferroptosis therapy in the treatment of PCa, providing a reference for future research and clinical application of ferroptosis.

## Supplementary information

Screenshot from email of all authors agreeing to change co-author

## Data Availability

The datasets used and analyzed during the current study are available from the corresponding author on reasonable request.
