# Supplementary material for: Ferroptosis landscape in prostate cancer from molecular and metabolic perspective
Source: Cell Death Discov. 2023 Apr 15;9:128. doi: 10.1038/s41420-023-01430-0 (PMC10105735; doi:10.1038/s41420-023-01430-0)
Supplement: Supplementary file 1 — Screenshot from email of all authors agreeing to change co-author [file 41420_2023_1430_MOESM1_ESM.pdf]

Re: Consultation on modifying co authors

"梁家鸣" <liangjiaming@tmu.edu.cn>

收件人: "蒋宁" <jiangning@tmu.edu.cn>

时 间: 2023-4-5 1:24:52

附 件:

Jiaming Liang

I agree it.

---- Replied Message ----

From	蒋宁<jiangning@tmu.edu.cn>
Date	04/05/2023 01:09
To	梁家鸣<liangjiaming@tmu.edu.cn>
Subject	Consultation on modifying co authors

5th Apr 2023

Dear Jiaming Liang

Due to the significant contributions made by Kun Yang and Yang Zhao in the revision process of the article "Ferroptosis landscape in prostate cancer from molecular and metabolic perspective" submitted to Cell Death Discovery. we have decided to add them to the co authors list. We now need to seek the agreement of other co authors. Please reply to your feedback.

Best regards,

Ning Jiang

Re: Consultation on modifying co authors

yihaoliao7777 <yihaoliao7777@163.com>

收件人: "jiangning@tmu.edu.cn" <jiangning@tmu.edu.cn>

Yihao Liao

时　间: 2023-4-5 12:26:59

附　件:

Dear professor Jiang:

Yes, I am informed and consented about the change of related author informations in the article "Ferroptosis landscape in prostate cancer from molecular and metabolic perspective" submitted to Cell Death Discovery.

Best regards,

Yihao Liao

廖义浩

16622080771

天津医科大学第二医院

300211

---- Replied Message ----

From	蒋宁<jiangning@tmu.edu.cn>
Date	4/5/2023 01:11
To	廖义浩<yihaoliao7777@163.com>
Subject	Consultation on modifying co authors

5th Apr 2023

Dear Yihao Liao

Due to the significant contributions made by Kun Yang and Yang Zhao in the revision process of the article "Ferroptosis landscape in prostate cancer from molecular and metabolic perspective" submitted to Cell Death Discovery. we have decided to add them to the co authors list. We now need to seek the agreement of other co authors. Please reply to your feedback.

Best regards,

Ning Jiang

回复：Consultation on modifying co authors

wangpu19940416 <wangpu19940416@126.com>

收件人：jiangning <jiangning@tmu.edu.cn>

时　间：2023-4-5 13:39:39

附　件：

Pu Wang

I agree

---- 回复的原邮件 ----

发件人	蒋宁<jiangning@tmu.edu.cn>
日期	2023年04月05日 01:12
收件人	wangpu19940416@126.com
主题	Consultation on modifying co authors

5th Apr 2023

Dear Pu Wang

Due to the significant contributions made by Kun Yang and Yang Zhao in the revision process of the article "Ferroptosis landscape in prostate cancer from molecular and metabolic perspective" submitted to Cell Death Discovery. we have decided to add them to the co authors list. We now need to seek the agreement of other co authors. Please reply to your feedback.

Best regards,

Ning Jiang

回复：Consultation on modifying co authors

"杨坤" <yangkundoct@163.com>

收件人：jiangning <jiangning@tmu.edu.cn>

时 间：2023-4-5 20:37:42

附 件：

Kun Yang

Thanks for the reminder, it's an honor to publish this article and an honor to be the author of this article.

best wishes

---- 回复的原邮件 ----

发件人	蒋宁<jiangning@tmu.edu.cn>
日期	2023年04月05日 20:20
收件人	yangkundoct@163.com
主题	Consultation on modifying co authors

5th Apr 2023

Dear Kun Yang

Due to the significant contributions made by Kun Yang and Yang Zhao in the revision process of the article "Ferroptosis landscape in prostate cancer from molecular and metabolic perspective" submitted to Cell Death Discovery. we have decided to add you to the co authors list. We now need to seek your agreement. Please reply to your feedback.

Best regards,

Ning Jiang

Re: Consultation on modifying co authors

15055152563 <15055152563@163.com>

收件人: "蒋宁" <jiangning@tmu.edu.cn>

时　间: 2023-4-5 15:16:50

附　件:

Youzhi Wang

I agree

---- Replied Message ----

From	蒋宁<jiangning@tmu.edu.cn>
Date	04/05/2023 01:15
To	15055152563@163.com<15055152563@163.com>
Subject	Consultation on modifying co authors

5th Apr 2023

Dear Youzhi Wang

Due to the significant contributions made by Kun Yang and Yang Zhao in the revision process of the article "Ferroptosis landscape in prostate cancer from molecular and metabolic perspective" submitted to Cell Death Discovery. we have decided to add them to the co authors list. We now need to seek the agreement of other co authors. Please reply to your feedback.

Best regards,

Ning Jiang

回复：Consultation on modifying co authors

"824797835@qq.com" <824797835@qq.com>

收件人：jiangning <jiangning@tmu.edu.cn>

时　间：2023-4-5 17:22:54

附　件：

Keke Wang

I have got that, I agree to add them to the co authors list.

824797835

邮箱：824797835@qq.com

---- 回复的原邮件 ----

发件人	蒋宁<jiangning@tmu.edu.cn>
日期	2023年04月05日 01:15
收件人	王可可<824797835@qq.com>
主题	Consultation on modifying co authors

5th Apr 2023

Dear Keke Wang

Due to the significant contributions made by Kun Yang and Yang Zhao in the revision process of the article "Ferroptosis landscape in prostate cancer from molecular and metabolic perspective" submitted to Cell Death Discovery. we have decided to add them to the co authors list. We now need to seek the agreement of other co authors. Please reply to your feedback.

Best regards,

Ning Jiang

回复：Consultation on modifying co authors

monkinsilence <18380793750@126.com>

收件人：jiangning <jiangning@tmu.edu.cn>

时　间：2023-4-5 12:34:15

附　件：

Boqiang Zhong

I agree with it.

---- 回复的原邮件 ----

发件人	蒋宁<jiangning@tmu.edu.cn>
日期	2023年04月05日 01:19
收件人	钟骏强<18380793750@126.com>
主题	Consultation on modifying co authors

5th Apr 2023

Dear Boqiang Zhong

Due to the significant contributions made by Kun Yang and Yang Zhao in the revision process of the article "Ferroptosis landscape in prostate cancer from molecular and metabolic perspective" submitted to Cell Death Discovery. we have decided to add them to the co authors list. We now need to seek the agreement of other co authors. Please reply to your feedback.

Best regards,

Ning Jiang

Re:Consultation on modifying co authors

"周典晟" <zhouds2021@163.com>

收件人: "蒋宁" <jiangning@tmu.edu.cn>

时　间: 2023-4-5 17:46:50

附　件:

Diansheng Zhou

Dear professor Jiang:

Yes, I am informed and consented about the change of related author informations in the article “Ferroptosis landscape in prostate cancer from molecular and metabolic perspective” submitted to Cell Death Discovery.

Best regards

周典晟

zhouds2021@163.com

---- Replied Message ----

From	蒋宁<jiangning@tmu.edu.cn>
Date	04/5/2023 01:17
To	周典晟<zhouds2021@163.com>
Subject	Consultation on modifying co authors

5th Apr 2023

Dear Diansheng Zhou

Due to the significant contributions made by Kun Yang and Yang Zhao in the revision process of the article "Ferroptosis landscape in prostate cancer from molecular and metabolic perspective" submitted to Cell Death Discovery. we have decided to add them to the co authors list. We now need to seek the agreement of other co authors. Please reply to your feedback.

Best regards,

Ning Jiang

Re: Consultation on modifying co authors

"20226020403 曹骞" <caoqian19990412@163.com>

收件人: "蒋宁" <jiangning@tmu.edu.cn>

时 间: 2023-4-5 14:48:10

附 件:

Qian Cao

I am informed and agree.

---- Replied Message ----

From	蒋宁<jiangning@tmu.edu.cn>
Date	04/05/2023 01:21
To	caoqian19990412@163.com<caoqian19990412@163.com>
Subject	Consultation on modifying co authors

5th Apr 2023

Dear Qian Cao

Due to the significant contributions made by Kun Yang and Yang Zhao in the revision process of the article "Ferroptosis landscape in prostate cancer from molecular and metabolic perspective" submitted to Cell Death Discovery. we have decided to add them to the co authors list. We now need to seek the agreement of other co authors. Please reply to your feedback.

Best regards,

Ning Jiang

Re: Consultation on modifying co authors

junboli13 <junboli13@163.com>

收件人："jiangning@tmu.edu.cn" <jiangning@tmu.edu.cn>

时　间：2023-4-5 13:03:58

附　件：

Junbo Li

agreement

junboli13

junboli13@163.com

---- Replied Message ----

From	蒋宁<jiangning@tmu.edu.cn>
Date	4/5/2023 01:22
To	junboli13@163.com<junboli13@163.com>
Subject	Consultation on modifying co authors

5th Apr 2023

Dear Junbo Li

Due to the significant contributions made by Kun Yang and Yang Zhao in the revision process of the article "Ferroptosis landscape in prostate cancer from molecular and metabolic perspective" submitted to Cell Death Discovery. we have decided to add them to the co authors list. We now need to seek the agreement of other co authors. Please reply to your feedback.

Best regards,

Ning Jiang

Re:Consultation on modifying co authors

"188725616@qq.com" <188725616@qq.com>

收件人: "蒋宁" <jiangning@tmu.edu.cn>

时 间: 2023-4-5 15:13:27

附 件:

Yang Zhao

Dear Pros. Ning Jiang,

Thank you for reaching out to me and offering me the opportunity to be a co-author on the article "Ferroptosis landscape in prostate cancer from molecular and metabolic perspective." I am honored to be recognized for my contributions to the revision process of the article.

I am happy to provide my agreement to be included as a co-author on this publication. Please let me know if there is any further information or documentation required from my end.

Thank you again for considering me for this opportunity. I look forward to collaborating with you and the other authors.

Best regards,

Yang Zhao

赵阳
188725616@qq.com

Original Email

Sender:"蒋宁"< jiangning@tmu.edu.cn >;

Sent Time:2023/4/5 1:23

To:"188725616@qq.com"< 188725616@qq.com >;

Subject:Consultation on modifying co authors

5th Apr 2023

Dear Yang Zhao

Due to the significant contributions made by Kun Yang and Yang Zhao in the revision process of the article "Ferroptosis landscape in prostate cancer from molecular and metabolic perspective" submitted to Cell Death Discovery. we have decided to add you to the co authors list. We now need to seek your agreement. Please reply to your feedback.

Best regards,

Ning Jiang
